# The neonatal transfer process through the lens of neonatologists at public hospitals in South Africa

**DOI:** 10.4102/hsag.v27i0.1617

**Published:** 2022-01-10

**Authors:** Pradeep Ashokcoomar†, Raisuyah Bhagwan

**Affiliations:** 1 Department of Emergency Medical Care, KwaZulu-Natal Department of Health’s Emergency Medical Services College, Durban, South Africa; 2 Department of Community Health Studies, Faculty of Health Sciences, Durban University of Technology, Durban, South Africa

**Keywords:** neonatologists, neonates, transfers, public hospitals, paramedics

## Abstract

**Background:**

Neonatal care is provided by various levels of healthcare facilities in South Africa. Intensive care for neonates is only provided at the higher levels, hence the need for transfers from lower-level to higher-level facilities (e.g. primary hospitals to tertiary hospitals) or across levels of facilities, particularly when life-threatening situations arise (e.g. cardiac deterioration, respiratory deterioration and desaturation).

**Aim:**

The aim of the study was to explore neonatologists’ views regarding the neonatal transfer process and to describe the preparedness of advanced life support (ALS) paramedics to undertake such transfers.

**Setting:**

The setting consisted of neonatologists from three provinces i.e. KwaZulu-Natal, Gauteng and Western cape.

**Method:**

A qualitative descriptive design was utilised in this study. Semistructured interviews were conducted on the public health hospitals in three provinces (*N* = 9; *n* = 3) with neonatologists (*N* = 7; *n* = 7) who were involved in the transfers of critically ill neonates. The process of thematic analysis was used.

**Results:**

The themes that emerged in this study were: an awareness of local contextual realities related to neonatal transfers, challenges evident within the context of neonatal transfers, decision-making around the transfer of ill neonates, ALS paramedic preparedness for transfers and good clinical governance

**Conclusion:**

The study found that there was a need to be aware of local contextual realities confronting neonatal transfers, a need for greater preparedness for paramedics to undertake these transfers, a need for a sound referral processes and a need for coordinated transfer effort between paramedics, hospital staff and transport team members for the successful transfer of critically ill neonates.

**Contribution:**

The findings highlight the challenges confronting the neonatal transfer process in South Africa through the lens of neonatologist at public hospitals. Hence, the study reinforces the preparedness and coordination of the transfer process, along with more efficient communication between paramedics, hospital staff and the transfer team.

## Introduction

A newborn infant, or neonate, is a child under 28 days of age, during which he or she is at the highest risk of dying (Negi et al. 2019). In sub-Saharan Africa, 1 in 36 children will die within the neonatal period (Masaba & Mmusi-Phetoe 2020). This is in comparison to high-income countries where the ratio is 1 in 333 (Ohunyan-Echichicya 2020). This emphasises the need to urgently consider how to reduce neonatal mortality rates in sub-Saharan Africa, as well as to reflect on the factors surrounding neonatal emergency care and transfers. Critically ill neonates require specialised hospital services. As neonatal intensive care exists only at a few facilities, critically ill neonates are generally transferred to higher-level healthcare facilities.

Emergency medical services (EMS) form the link between these healthcare facilities and are an extension of the intensive care unit (ICU). In order to ensure the best possible services, trained health professionals in infant healthcare are essential. Gilpin and Hancock (2016) stated that acknowledging the potentially hazardous environment of the pre-hospital setting outside the neonatal intensive care unit (NICU), reinforces the need for the transfer process to be organised and efficient. The practice of inter-healthcare facility transportation of critically ill neonates continues to expand and has evolved into mobile ICUs in developed countries capable of delivering state-of-the-art practices for critical care outside the NICU, thus maintaining or improving the continuum of care. An expert level of care underpinned by sound organisational structures and processes is therefore essential. This includes personnel with the appropriate knowledge, skills and attitudes to deal with the added challenges of a neonatal transfer and the necessary specialised equipment and supplies (Kronforst 2016). It is against this backdrop that this study sought to understand neonatologists’ views with regard to the transfer process and the preparedness of paramedics for these transfers.

When failed or ineffective systems exist for transporting critically ill neonates, the risk of morbidity and mortality increases. The main goals of neonatal-paediatric transport teams are early stabilisation and the initiation of advanced care at the referring institution, with a continuation of critical care therapies and monitoring en route, so as to improve the safety of the transport and the neonates’ outcome. This requires competence and skill of the paramedics involved in these transfers. In this vein, Disque (2021) argued that the neonate’s condition should not deteriorate as a result of preventable issues during the pre-transport and transport process.

Despite the tendency to expedite the transfer process, it is often forgotten that in an emergency, speed is no substitute for the time invested in resuscitating and stabilising the neonate before leaving the referring facility. There is rarely a need for haste and panic, as this often results in morbidity or mortality. Numerous studies, both in resource-limited (Ashokcoomar & Naidoo 2016; Henry & Trotman 2017) and developed settings (Fenton & Leslie 2009; Fetus & Newborn Committee 2015; Gilpin & Hancock 2016) emphasise this point. All these studies found that the level of training of transfer teams to perform high-level interventions for stabilising the neonate before transfer and the continuous advanced care and monitoring during the transfer reduces the risk of adverse events. These studies also found that despite pre-transport stabilisation and packaging and the best preparation, neonates may deteriorate in transit. The transport team should therefore be skilled and equipped to clinically manage these situations.

High risk neonates who are transferred have a significantly greater risk of death because of severe respiratory distress syndrome, intraventricular haemorrhage and nosocomial infections. These neonates are often critically ill and their outcomes hinge on the effectiveness of the transport system (Sundrani et al. 2019). This calls for a greater scrutiny of the transport system in South Africa.

Internationally, neonatal transport is conducted by a team of 2–3 clinicians, which may include nurses, respiratory therapists, neonatologists, neonatal nurse practitioners or paramedics. Generally, there are two models of the National Task Team, namely unit-based teams and dedicated teams. *Unit-*based teams are composed of the NICU and healthcare professionals. The responsibilities of the latter include both bedside patient care and attending to transport calls. This model is usually adopted in hospitals where there is a shortage of staff (Soliman et al. 2019). *Dedicated teams*, on the other hand, comprise members whose primary responsibility is to transport neonates without any bedside patient care assignments in the NICU (Lee, Khang & Lim 2019). However, they can provide support and help to NICU staff in between calls by performing procedures, resuscitations and transporting admitted neonates from the NICU to other departments within the hospital for procedures such as surgical operations or diagnostic radiological investigations, if needed. This model requires special training and clinical competencies (Soliman et al. 2019). Despite staff shortages, what is evident is the need for dedicated transport teams as their primary responsibility would be to transfer critically ill neonates.

Despite advancements in the field of paediatrics in the 1960s and 1970s and the maturation of the adult emergency medical system in the United States, the transportation of critically ill children remained a serious challenge. Neonates and children were subjected to high-risk environments, limited resources and poorly trained transport personnel, resulting in significant morbidity and often death before arrival at the accepting centre. Hospital-based transport teams arose out of necessity, using resources and clinicians from tertiary hospitals’ neonatal units to retrieve infants (Campbell & Dadiz 2016).

The facility of neonatal transport is a major gap in holistic neonate care in our country. The successful transport of the neonate depends largely on the mode of transport, trained transport team, adequate equipment, appropriate drugs and effective communication. At the same time, it is well known that the transport of the neonate by a skilled organised team reduces neonatal mortality and morbidity (Hapani, Berwal & Dave 2019). Most neonates are transported in private or public vehicles without any pre-referral stabilisation or care during transport. Many of the neonates transported in this way are hypothermic, cyanotic and hypoglycaemic and a large number (75%) has serious clinical complications (Rakholia et al. 2014). These complications further increase the morbidity and mortality of these ill neonates. A reduction in infant mortality rate (IMR) can only be achieved by improving the neonatal transport facilities (Shalini, Nikhila & Alimelu 2017).

Transfers of neonates in other developed countries are structured and use well-developed regionalised programmes with a cadre of appropriately skilled human resources (McEvoy et al. 2017). They operate under advanced medical directives with access to telephone clinical advice, preferably from senior physicians with specific neonatal and transport expertise. There is considerable diversity in terms of those who undertake the neonatal transfer. These include combinations of physicians, paediatricians, neonatologists, respiratory therapists, anaesthetists, nurses and paramedics. Although there are no published studies to date comparing professions, studies have recommended the use of specifically trained personnel for critically ill neonatal transfers, as they are the key to safe transport, irrespective of the profession (Chang et al. 2015; Gente et al. 2015; Kue et al. 2011).

Teamwork is essential for providing care and therefore, the process of improving teamwork has received top priority (Buljac-Samardzic, Doekhie & Van Wijngaarden 2020). A wide range of studies have indicated a positive effect of team training on performance outcomes within diverse healthcare setting (Hughes et al. 2016; Murphy, Curtis & McCloughen 2016; Tan et al. 2014). Results from 20 studies reviewed by Costar and Hall (2020) further revealed that well-implemented team training programmes positively impact the transfer of teamwork skills, improve clinical processes and increase patient safety (Hughes et al. 2016). Kronforst (2016) reported that in the United Kingdom, 75% of children transferred experienced adverse events primarily because of unreliable processes and non-specialised teams undertaking the transfer. According to Akula et al. (2020), this unpredictability can be dealt with by engaging in teamwork, gathering experience with staff at referral hospitals, planning for a wide variety of circumstances, specialised training, debriefing after events and implementing quality improvement strategies.

Likewise, a systematic review of published electronic sources from 1998 to 2009 by Fanara et al. (2010) found that transfer systems should be evaluated on a regular basis to ensure the quality of patient management, especially with the critically ill patient. This was also supported by Romanzeira and Sarinho (2015).

Similarly, Kue et al. (2011) reported a reduction in adverse events ranging from 60% to 70% when using a dedicated unit for the transportation of neonates. Studies have found that most adverse events occurred when trained, dedicated transport providers were not used, therefore ‘it could be inferred that the transport itself requires an additional set of knowledge and skills beyond that of standard critical care in the ICU setting’ (Kue et al. 2011:2).

## Methods

### Study design

A qualitative descriptive design was used to explore neonatologists’ views regarding the neonatal transfer process and the preparedness of advanced life support (ALS) paramedics to undertake such transfers.

### Setting

Neonatologists from across the county who work in the public health sector were invited to participate. There are very few neonatologists in South Africa, therefore the availability of this sample was challenging. Those included were located in the provinces of KwaZulu-Natal, Gauteng and the Western Cape.

### Study population and sampling strategy

Neonatologists who worked at public health hospitals were invited to participate. Invitations were sent out to neonatologists located at these facilities in the provinces of KwaZulu-Natal, Gauteng and the Western Cape (*N* = 9; *n* = 3). Data were collected from (*N* = 7; *n* = 7) neonatologists.

Purposive sampling was used for both the public healthcare hospitals and neonatologists. There are a limited number of neonatologists employed at the public health hospitals. Most of the participating neonatologists were found to be heads of the NICU at these hospitals.

### Data collection

Data were collected using in-depth semi-structured interviews guided by a predetermined interview schedule. The advantage of semi-structured interviews is their capacity to invite the interviewees’ experiences and encourage broader dialogue, whilst remaining attentive to the focus of the research. No pilot study of the schedule was undertaken given the limited number of neonatologists in practice. However, the interview schedule was piloted with ALS paramedics and EMS lecturers. The researcher identified the neonatologists working in the public sector and they were sent an invitation, asking them to participate. A letter of information and informed consent was signed voluntarily by each participant prior to conducting the interviews. The interviews lasted between 60 min and 90 min and were audio recorded with the written consent of the participants.

The interview schedule consisted of the following questions:

- What are your thoughts on the current inter-healthcare facility transfer for critically ill neonates?
- What specific knowledge should the ALS practitioner have about the transfer process?
- How are ALS paramedics prepared to deal with the critically ill neonates?
- What interventions can be put in place to develop a more effective transfer programme?
- What are the psychosocial needs of family members during the transfer?

The interviews elicited rich and relevant data. Data collection stopped after saturation was achieved with seven neonatologists.

### Data analysis

The data were analysed using the steps of thematic analysis as outlined by Braun, Clarke and Hayfield (2019). Thematic analysis allowed the researcher to make sense of collective meanings and experiences. This helped to organise and reduce the data into themes. A preliminary coding scheme was generated, which served as a template for the data analysis. Similar themes and recurring patterns in the data were linked together and the contrasts and differences identified (Guba 1994).

### Trustworthiness

To enhance the trustworthiness of this study, the researcher used Guba’s model (1994). This model provides four criteria to ascertain rigour in qualitative studies, namely credibility, dependability, conformability and transferability. Strategies used were (1) member checking, (2) the triangulation of data (multiple data sources were used, as ALS paramedics and emergency medical care [EMC] academics were also interviewed, although this study reports solely on the views of neonatologists), (3) maintaining an audit trail and (4) keeping a reflective journal. This ensured the trustworthiness of the data and reflexivity of the researcher. In addition, (5) an expert evaluation committee was also used to validate the findings made. The latter were independent experts who reached consensus around the themes generated. Moreover a co-coder was used, which strengthened the trustworthiness of the findings.

### Ethical considerations

Ethical clearance for this study was obtained from the Institutional Research Ethics Committee (IREC) of the Durban University of Technology. The study was allocated ethical clearance number IREC 093/15. Before the commencement of the data collection process, all the participants were informed verbally and by letter that their participation was completely voluntary; that they could withdraw from the study at any time without repercussions; that anonymity would strictly be maintained and that their identifying details would be kept confidential. They were also informed that there would be no financial benefits for participating in the research, and the study’s findings would be disseminated through publications, which would potentially facilitate an improved transfer process.

## Results

There were five themes that emerged in the data, namely an awareness of local contextual realities related to transfers (Theme 1), challenges evident within the context of neonatal transfers (Theme 2), decision making around the transfer of ill neonates (Theme 3), ALS paramedic preparedness for transfers (Theme 4) and good clinical governance (Theme 5). Each of the five themes is discussed in more detail in Table 1.

**TABLE 1:** Table representing main themes that emerged from the study.

Number	Theme
1.	An awareness of local contextual realities related to transfers
2.	Challenges evident within the context of neonatal transfers
3.	Decision making around the transfer of ill neonates
4.	Advanced life support paramedic preparedness for transfers
5.	Good clinical governance

### Theme 1: An awareness of local contextual realities related to transfers

The neonatologists interviewed highlighted the need for a greater awareness of local contextual realities unique to developing countries such as South Africa. One participant particularly emphasised that the best practices in developed countries would not work in resource-poor countries such as South Africa.

‘Let[*s*] do things that suits us, what is beneficial and realistic in our setting. Best practice in first world countries does not necessarily mean best practice in resource-poor countries, like ours.’ (N7)

‘There is much need for improvement in the intensive care neonatal transfers; especially in the public sector, there are too many variations, unplanned and disorganised transfers with no proper structures in place, and as a result, there are long delays, [*as well as*] logistical and technical issues. These add further complications to a baby who already has critical complications.’ (N5)

‘Communication and coordination are poor, and this is one of the key importance of a transfer system for the best interest of the patient and service delivery.’ (N6)

‘There is a lack of communication and sometimes miscommunication, so communication needs a lot of attention as it is the core of transfer. Everyone dealing with the patient needs to know what is going on.’ (N1)

This suggests the need to scrutinise neonatal transfer dynamics more closely with a view to developing services in relation to challenges that resource-poor environments bring. Safe transport in resource-poor communities is premised on several factors, such as the ambulance as a means of final transport, pre-referral treatment or stabilisation, and ambulance accompanying health personnel and prior communication from referring centres (Negi et al. 2019).

One participant highlighted that local transfers are disorganised and the lack of structure compromises a neonate who is already critically ill. Another reported that neonatal transfers in South Africa have evolved in a largely unplanned manner with little or no formal structure, which has resulted in variations of practice, depending on different geographical regions and the availability of resources. Whilst it may not always be possible to have total consistency, certain protocols should be in place, regardless of the geographical area in South Africa. For a start, a dedicated organisational structure and a team that coordinates and oversees the transfer should be at the heart of all transfers. As one participant reported, communication amongst all those involved in the transfer process was seen as the most important factor related to a successful transfer. O’Mahony and Woodward (2018) emphasised that it is vital to ensure that relevant skills and therapy are present and administered throughout the transfer process, all the way from referral call through definitive placement, whilst also focusing on seamless transition at each point of care. Clear and pertinent communication must be provided by the team, with the consultation of the patient’s physicians and documentation of their advice, interventions and activities that enable appropriate patient care.

### Theme 2: Challenges evident within the context of neonatal transfers

The neonatologists in the sample reported that the equipment required for neonatal transfers posed a significant problem. They indicated that equipment was either unavailable or malfunctioning. A lack of regular service of the equipment, specifically cold incubators and battery failures are some of the challenges confronting ALS paramedics involved in neonatal transfers. The following verbatim excerpts from the participants’ interviews support this theme:

‘Technical and logistics issues with equipment are a huge concern that we are currently facing. There are always problems with equipment, it’s either they don’t work or it’s not available. I know that equipment was procured but it comes down to poor management of these equipment because they are not serviced and well-kept, especially the incubators, ventilators and syringe drivers.’ (N1)

‘They are borrowing equipment for the transfer, which is the incorrect thing to do. Sometimes the paramedics come without dated equipment, faulty equipment, cold incubators and battery failures. So the problems with EMS equipment are endless.’ (N3)

‘Having a critical neonate in an ambulance on a portable ventilator for a long period is not advisable, simply because of the potential dangers of a portable ventilator, mainly barotrauma, and all the risk of the ambulance environment. Therefore, long distance transfer must be airlifted to speed up the process.’ (N7)

‘Waiting for long periods for a baby to arrive is problematic, not only for the baby but it has a ripple effect on the whole system. Doctors and nurses have to wait for the baby, we are very busy, and we cannot just sit around and wait. Delays must be prevented; it is the responsibility of the EMS.’ (N5)

One participant also pointed out the acute shortage of appropriate equipment, saying that paramedics have to borrow equipment, which is not good practice.

Another neonatologist reiterated the dangers of using portable ventilators for long periods because of the risk of barotrauma in the ambulance environment. He suggested the need to consider air transfers when the transfer is over a greater geographical distance.

Another participant echoed the need for time delays to be addressed, saying that this is detrimental to the neonate. He added that often doctors and nurses had to wait, and it is crucial that EMS be responsible in terms of preventing delays. It was Gilpin and Hancock (2016) who stated that a longer duration of transfer was considered as an independent risk factor associated with adverse events during neonatal transfers. A study performed by Mori et al. (2007) accentuated the plight of ill neonates, indicating that those transported for more than 90 min were found to have a high risk of experiencing life-threatening adverse events. As such, Kumar et al. (2010) recommended that any transfer of more than 90 min must be undertaken by air ambulance, as suggested by one of the participants. The care delivered in the transport environment is distinct from the NICU and is significantly affected by technical and logistical issues such as equipment, the neonatal transfer team and travel distance. Thus, it is essential to factor in these technical and logistical issues as part of quality indicators (Lee et al. 2019). Neonatal patients have longer stabilisation times than paediatric patients (median stabilisation time of 80 min vs. 45 min) and neonates with a higher severity of illness (defined as ventilated and on inotropes) have longer stabilisation times compared with non-ventilated neonates (median stabilisation time 125 min vs. 63 min) (Levine et al. 2016). Hence, instead of minimising the stabilisation times, a major goal would be to expedite the arrival of the neonatal transfer team at the referral site (response time) so that the infant can benefit from their advanced skills and the arrival of the infant at the destination hospital for definitive care (Lee et al. 2019).

### Theme 3: Decision making around the transfer of ill neonates

The data emerging under Theme 3 reflected the importance of sound decision making before a neonatal transfer is conducted. The issues given prominence related to the importance of considering the availability of resources, and decision making should include the receiving doctor. Some of the participants’ views on this theme are captured in the following quotes:

‘Do not transfer an unstable baby because you are just going to make a bad situation worse. I do understand that at times there is a degree of intimidation to transfer an unstable neonate; in that case, the paramedic must phone the receiving doctor and the consultant and discuss the matter, let them get involved in this decision.’ (N3)

‘We also need to keep in mind the context we are in, there is always resource issues, and if you leave the baby at a low level hospital, the baby will die, for example, i[*f*] unstable, and the referring hospital do[*es*] not have a ventilator, you cannot leave the baby there, the baby will die. So in that case, speak to the relevant people, [*get*] the doctors involved, and the transfer team, and take an informed decision, but it must be clear that the baby is unstable.’ (N6)

Some neonatologists pointed out that paramedics are at times intimidated to transfer unstable babies. They again pointed out the challenges of working in resource deprived environments, and if the neonate had to remain at a hospital without a ventilator, he or she could die. Two neonatologists called for a joint discussion between the ALS paramedic, referring and receiving doctors or the entire transfer team, before decisions are made with respect to transfers. Two neonatologists stated that unstable neonates should in principle not be transferred. Similarly, a hospital-based study of 500 neonates admitted to the NICU at Niloufer Hospital for Children found that low birth weight, prematurity, pre-referral stabilisation, untrained person accompanying the neonate, lack of awareness of danger signs and duration of transport > 1 h, were significant predictors of neonatal mortality. This emphasises the need for proper regionalisation of peripheral care and appropriate pre-referral stabilisation and transport care of neonates (Shalini et al. 2017).

Other scholars have highlighted the stressors associated with ambulance transfers, namely excessive noise, vibration, poor lighting and limited space (Lorch et al. 2012). These stressors can directly or indirectly influence the physiological condition of the neonate, which can cascade into a life-threatening situation during the transfer journey. Adequate preparation and stabilisation of the neonate is therefore crucial prior to the transfer journey. Most important, however, is the assessment of risks associated with the transfer, which calls for sound clinical competence in making such decisions. An informed joint decision by the referring, transporting and receiving personnel is critical to ensuring that no one can be held accountable for any adverse events encountered during transportation (Kage & Akuma 2012). In addition, paramedics should not be intimidated by the real or perceived power of other healthcare providers, and should engage in joint decision making when it comes to unstable babies.

The ideal environment for pre-hospital stabilisation, as indicated by Gilpin and Hancock (2016), is not only the NICU but also emergency departments because of the availability of resuscitation equipment and senior specialist input. Stabilisation prior to transport is important as it allows the neonate to remain in a stable condition for a longer period of time, thus reducing transfer stress, as interventions are difficult to administer during transfers. Optimal pre-transport stabilisation results in a lesser chance of the neonate deteriorating during transfer. Inadequate pre-transfer preparation was echoed by Lim and Ratnavel (2008) who found that human-related adverse events accounted for 67% of all adverse events, with 50% thereof occurring as a result of poor preparation and stabilisation.

### Theme 4: Advanced life support paramedic preparedness for transfers

The fourth theme that emerged from the data reflected the importance of the preparedness of ALS paramedics before they could undertake neonatal transfers and the need for cross-training opportunities. In this regard, the participants remarked as follows:

‘For a paramedic to be prepared to manage an intensive care neonate, they have to have the current and continuous correct education and training, with the right attitude.’ (N7)

‘Preparedness will have to be the correct education. Paramedics have education in critical care; they have a qualification, but it needs to be continuously refreshed; they need to know what does the latest evidence say, keep their skills sharp, continuous practice. So it’s not just about getting a qualification, it [is] also about maintaining the knowledge and skills, that how they can be prepared.’ (N3)

‘I think that cross-training could be beneficial. Paramedics will learn a lot from this process, and if the NICU doctors spent time on the ambulances, it would certainly give us insight into the challenges. So, from that point of view, I think it could be beneficial because NICU interviews with ALS paramedics doctors will understand what a transfer is all about and what is going on; and then I definitely think that there’s benefit in terms of the paramedic spending time in NICU, because they will understand what’s going on in the hospital.’ (N1)

Neonatologists in the sample were of the view that appropriate educational preparedness was required for ALS paramedics to work in this specialised area of EMC. One participant expressed the need for such education to be specifically tailored to neonatal clinical emergencies and transfers. In addition to the fact that such educational preparedness needed to be underpinned by specialised knowledge in this field, they also emphasised the need for continuous professional training in order to keep abreast of contemporary knowledge and skills in the field.

Whilst neonatologists emphasised appropriate knowledge, they also underlined the importance of being competent in relation to the specialist skills required for clinical interventions on neonates. Several traditional skills have been identified as essential for transport team members. These include airway management, intravenous (IV) and intraosseous access, umbilical venous catheterisation, chest tube or thoracostomy needle placement for pneumothorax and defibrillation (King & Woodard 2002). Airway management and intubation are key skills to master for transport team members, because up to 20% of infants require intubation during neonatal transport (Campbell & Dadiz 2016).

There have been several areas detailed in the literature. These include teamwork skills, communication, environmental awareness, decision making and patient symptoms for transport teams working in multiple environments under stressful conditions. Pre-hospital, ambulance, hospital units and aircraft environments present unique challenges to experienced healthcare professionals (Reimer & Moore 2010). Curricula that address non-technical skills have resulted in improved patient care (Campbell & Dadiz 2016).

One of the most important aspects emerging from the data was the need to consider cross-training. Neonatologists affirmed that paramedics could learn a lot from spending time in NICUs, where they could learn from the neonatologists themselves. They could also understand the dynamics and processes of NICUs. It was also important that participants believed that they themselves would benefit from spending time in the ambulances, where they could develop insight into the transfer challenges paramedics faced.

### Theme 5: Good clinical governance

The need for regular audits and feedback to assess the transfer team and the referring and receiving facilities performance was also identified by neonatologists. In this regard, they had the following to say:

‘There has to be a good system in place for regular audits and feedbacks. In my view, this does not exist, only when there is a service complaint, then we go and look for the problem. Neonatal transfers are teamwork between the hospitals and ambulance service; therefore, there needs to be feedback [and] audits to see what is going on, what should happen, why it’s not happening. It’s an improvement mechanism.’ (N3)

‘I definitely feel that there should be some clinical governance. We must professionally appraise our system by quality assuring it through audits, compare our system with others. There must always be room for improvement.’ (N3)

The neonatologists expressed that good clinical governance was important, particularly regular audits and feedback with respect to the transfer process. One participant said that neonatal transfers were not the sole responsibility of paramedics but involved a joint, shared team process between the hospital and the ambulance service. He added that the cornerstone of good clinical governance for neonatal transfers is regular audits and feedback, so as to be aware of what was going on, to understand why certain things are not happening and to improve the process. He indicated that such audits do not occur regularly, but exist only when there is a service complaint. Another participant supported the notion of quality assurance processes to appraise the current transfer system. More importantly, he suggested the need to compare the South African system with other transfer systems to enable improvement.

## Discussion

The study found that neonatal transfers occur in a relatively disorganised way. These findings were supported by several researchers who brought to light that there are few standardised protocols for the transfer and transport of a sick neonate in low-income countries (Negi et al. 2019; O’Mahony & Woodward 2018; Tette et al. 2020). Where transportation is available, the lack of proper emergency care on the way to more specialised facilities puts many neonates’ lives at risk. This was evidenced through the relative unpreparedness of ALS paramedics to deal specifically with neonatal transfers. The essence of neonatal transport medicine is to keep the neonate stable; improve their clinical status and ensure that they have not deteriorated further upon arrival at the receiving hospital. There are several concerns that must be addressed through the organisational structures of an inter-healthcare transfer programme, particularly communication, staff, equipment and monitoring. These are deserving of greater consideration to improve the neonatal transfer system in South Africa.

The study found the need for specialised training of ALS paramedics in neonatal transfers and ongoing capacitation to ensure that they are prepared for all neonatal emergencies. This was supported in the literature for personnel with the appropriate knowledge, skills and attitude to deal with the added challenges of the transfer and the necessary specialised equipment and supplies (Fortune, Parkins & Playfor 2017). The outcome of a critically ill neonate during the transfer depends largely on the degree of technology, the expertise of personnel and the training programmes undergone by the transfer teams.

Another critical issue raised was the acute absence of relevant equipment and the fact that when it was available, it was malfunctioning. Hence, whilst paramedic expertise was a factor, the requisite equipment was critical to supporting paramedic expertise. These findings correlated with those of Sundrani et al. (2019) who indicated that improving transport conditions of sick neonates should include the prevention of hypothermia by ensuring the availability of incubators, radiant heaters, heated water-filled mattresses and kangaroo mother care. They added that neonatal hypoglycaemia causes long-term neurological sequelae, which can be prevented by giving IV boluses before transport, maintaining IV access and continuous fluid infusion during transport and feeding of a stable neonate. This requires the availability of equipment and other medical devices or treatment to support a safer transfer.

A further issue highlighted was the need for a coordinated approach to the transfer between all stakeholders involved from the referring to the receiving hospital. When a neonate infant is referred, transport may interfere with several aspects of his or her homeostasis, such as thermo-regulation, metabolic stability, fluid and electrolyte balance and cardiorespiratory status, amongst others. A successful transport system thus involves two transitions of care of neonates, from the referring hospital staff to the transport team and from the transport team staff to the accepting hospital staff. Transportation of the sick or preterm babies to a centre with expertise and facilities for the provision of multi-organ intensive care has been shown to improve outcomes (Sundrani et al. 2019). It is within the context of proper coordination that sound decisions can be made through consultation with all stakeholders involved in the process.

Finally, the study found the need for rigorous clinical governance related to the transfer process. It is important to understand the dynamics within the entire transfer process, from the time of the initial request for the transfer, till the transfer team returns to their base, and there should be cognisance of technical, logistical and clinical data. This would ultimately serve to monitor the transfer process and highlight the shortcomings that require attention. Given that transfers involve a multidisciplinary team, it is also crucial that feedback be obtained from all the stakeholders involved to ensure that their expertise and knowledge is harnessed in terms of the transfer process.

### Limitation

There are very few neonatologists in South Africa. Hence, securing a larger sample from the public sector proved challenging. Those included were located only in the provinces of KZN, Gauteng and the Western Cape. This may therefore not reflect the views of all neonatologists and all the issues at public hospitals nationally. The information richness gleaned from those interviewed however shed valuable light on the challenges confronting the neonatal transfer process.

### Recommendations

Further research into the contextual local realities of the neonatal transfer process in South Africa is recommended. It is crucial to understand the infrastructure-related challenges and issues pertaining to specialised staff so that a safe neonatal transfer process that addresses the issues in South Africa occurs. The need for specialised equipment and equipment that is functioning optimally and is readily available as transfers occur is also crucial. Hence, a further recommendation is the need for an audit of equipment, which is required. The other most important recommendation is the educational institutions that prepare paramedics for EMC, integrate knowledge and clinical skills training with regard to neonatal transfers in their educational programmes.

## Conclusion

The study shed valuable light on the neonatal transfer process in South Africa through the lens of neonatologists at public hospitals. They drew attention to the need to be aware of the constraints brought on by the contextual realities of developing countries such as South Africa. More importantly, they suggested the need for a closer scrutiny of the transfer process in terms of equipment-related challenges and the need for specialised preparedness of paramedics to cope with the intense nature of these transfers. In addition, the study documented the need for sound referral processes and a coordinated transfer effort between paramedics, hospital staff and transport team members.
